# Prognostic factors related to sequelae in childhood bacterial meningitis: Data from a Greek meningitis registry

**DOI:** 10.1186/1471-2334-11-214

**Published:** 2011-08-10

**Authors:** Vasiliki A Vasilopoulou, Maria Karanika, Kalliopi Theodoridou, Antonios T Katsioulis, Maria N Theodoridou, Christos S Hadjichristodoulou

**Affiliations:** 1 Department of Hygiene and Epidemiology, University of Thessaly, Larissa, 41221, Greece; 2 First Department of Pediatrics, Aghia Sofia Children's Hospital, University of Athens, 11530, Greece

## Abstract

**Background:**

Bacterial meningitis (BM) is a life-threatening disease, often related with serious complications and sequelae. Infants and children who survive bacterial meningitis often suffer neurological and other sequelae.

**Methods:**

A total of 2,477 patients aged 1 month to 14 years old hospitalized in a Children's Hospital in Greece diagnosed with acute bacterial meningitis were collected through a Meningitis Registry, from 1974 to 2005. Clinical, laboratory and other parameters (sex, age, pathogen, duration of symptoms before and after admission) were evaluated through univariate and multivariate analysis with regard to sequelae. Analysis of acute complications were also studied but not included in the final model.

**Results:**

The rate of acute complications (arthritis and/or subdural effusion) was estimated at 6.8% (152 out of 2,251 patients, 95%CI 5.8-7.9) while the rate of sequelae (severe hearing loss, ventriculitis, hydrocephalus or seizure disorder) among survivors was estimated at 3.3% (73 out of 2,207 patients, 95%CI 2.6-4.2). Risk factors on admission associated with sequelae included seizures, absence of hemorrhagic rash, low CSF glucose, high CSF protein and the etiology of meningitis. A combination of significant prognostic factors including presence of seizures, low CSF glucose, high CSF protein, positive blood culture and absence of petechiae on admission presented an absolute risk of sequelae of 41.7% (95%CI 15.2-72.3).

**Conclusions:**

A combination of prognostic factors of sequelae in childhood BM may be of value in selecting patients for more intensive therapy and in identifying possible candidates for new treatment strategies.

## Background

Bacterial meningitis (BM) is a life-threatening disease, often related with serious complications and sequelae. During the last decades the disease epidemiology has changed dramatically in the countries that adopted the conjugate vaccines against *Haemophilus influenza *type b (Hib), *Neisseria meningitidis *and *Streptococcus pneumoniae*. However the impact of these vaccines on the disease epidemiology needs to be further investigated during the next few years. Infants and children who survive bacterial meningitis suffer neurological and other sequelae, including hearing impairment, adversely affecting academic performance [1]. The incidence rate (IR) of residual abnormalities in post meningitic children is approximately 15% (with a range of 10% to 30%) [2]. As previously reported the mean annual incidence rate of BM in Greece throughout a period of 32 years has been estimated to be 16.9/100,000 children, for meningococcal meningitis 8.9/100,000 children, for pneumococcal meningitis 1.3/100,000 children, for Hib meningitis before conjugate vaccine 2.5 per 100,000 and for Hib meningitis after conjugate vaccine 0.4 per 100,000 children [3].

Knowledge about factors associated with poor prognosis could be valuable in selecting patients for more intensive monitoring and treatment, in order to further improve outcome. Several risk factors associated with sequelae in childhood BM have been identified in previous studies, such as the specific organism causing the disease, duration of illness before admission, presence of convulsions, focal neurologic deficits, depressed level of consciousness or absence of petechiae on admission and low cerebrospinal fluid (CSF) glucose [4-6]. However, findings of various studies are conflicting or inconclusive, either due to the limited number of patients or due to differences in statistical analysis (univariate *vs *multivariate analysis).

The aim of this study was to present among others the clinical and laboratory characteristics of a significantly large population of children presenting with BM, and to identify independent prognostic factors for sequelae, by applying a multivariable analysis approach.

## Methods

### Ethics Statement

Data currently presented has been derived from the Meningitis Registry (MR) of the Infectious Diseases Department of Aghia Sophia Children's Hospital in Athens, Greece. The Registry Form included approximately 50 parameters that were recorded by the attending physician for each patient including demographic data, clinical and laboratory data on admission and during the hospital stay, treatment information, discharge diagnosis, outcome and sequelae. Follow up visits were conducted up to 3 months after discharge in surviving patients and all additional information was recorded. The detailed methodology concerning the study setting, data collection, inclusion and exclusion criteria have been described elsewhere [3]. This study was approved by the ethics committee of Aghia Sofia Children's Hospital. Patient consent was not required since the data presented in this study did not relate to any person or persons.

This study presents 32 years of cumulative data concerning clinical presentation, laboratory findings and prognostic factors of bacterial meningitis from patients aged one month to 14 years of age. Data related to treatment will be discussed elsewhere.

### Definitions

A "*Bacterial Meningitis case*" was defined as any patient treated in the Infectious Diseases Department of Aghia Sophia Children's Hospital from January 1^st ^1974 until December 31st 2005, who was aged 1 month to 14 years old and was diagnosed with bacterial meningitis [3].

The "*Study population" *included both "*probable" *and "*confirmed" *cases.

*"Probable bacterial meningitis" *cases were defined - according to World Health Organization (WHO) criteria as those presenting clinical symptoms of meningitis (i.e. fever, headache, stiff neck, bulging fontanelle or altered mental status) and CSF with an elevated protein (> 100 mg/dl), decreased glucose (< 40 mg/dl) or leukocytosis (> 100 WBC/mm3) with at least 80% neutrophils and lacking an identifiable bacterial pathogen [7,8].

"*Confirmed bacterial meningitis" *cases were defined according to WHO case definition criteria [7,8]: children presenting with clinical symptoms of meningitis (i.e. fever, headache, stiff neck, bulging fontanelle or mental status changes) and identification of bacteria directly (by culture or PCR from blood or CSF, or by culture from the petechial lesions), or indirectly (by latex test, countercurrent immunoelectrophoresis, Phadebact (latex agglutination), or Gram stain smear of blood or CSF). The "*Confirmed bacterial meningitis" *cases were further divided into the following groups, representing the most commonly isolated pathogens: *Neisseria meningitidis*, Streptococcus *pneumoniae*, *Haemophilus influenzae *type b and a fourth group "confirmed bacterial meningitis due to other bacteria" including all other isolated pathogens.

*Exclusion Criteria*: Patients with recurrent meningitis due to structural defects of the central nervous system, or with Tuberculous meningitis were excluded from the analysis. Patients less than one month old were also excluded from the study.

*"Fever" *was defined as body temperature ≥ 38°C.

*"Shock" *was defined as a systolic blood pressure that was < 2 Standard Deviations (SD) of the age-related mean value or severely decreased peripheral perfusion during physical examination.

*"Coma" *was defined as Glascow Coma Scale (GCS) < 8 or a state of profound unconsciousness in which the child was incapable of sensing or responding to external stimuli.

*"Acute complications" *were defined as the presence of arthritis or subdural effusion. *"Sequelae" *were defined as the presence of severe hearing loss, ventriculitis, hydrocephalus or seizure disorder, during the follow up period of 3 months.

*"Arthritis" *was defined as the presence of pain, swelling and restriction of movement in a joint.

The term *"severe hearing loss" *was applied to a hearing threshold of ≥ 80 dB as assessed by an audiologist.

*"Seizure disorder" *was defined as any convulsive disorder of any type that did not exist before the onset of BM and was present during and after hospitalization.

"*Bulging fontanelle*" and "*grunting*" were examined in children less than two years old.

"*Meningeal signs*" were examined in children greater than or equal to one year of age.

"*Headache*" was examined in children greater than or equal to two years old.

### Study periods

For practical reasons the study was divided by date of admission into three time intervals: period A (1974-1984) representing the pre Hib era, period B (1985-1994) representing the introduction of Hib vaccine and period C (1995-2005) representing the post Hib vaccine era. The Hib conjugate vaccine was introduced in Greece in mid 1994, as a result period C represents the era after the Hib conjugate vaccine was widely used. The heptavalent pneumococcal vaccine was introduced in 2004.

### Data analysis

Statistical analysis was performed by using the EPI-INFO (version 3.4.3 - CDC - Atlanta) and SPSS (version 15.0 - Chicago) software. Chi-square test or Fisher's exact test were used to compare qualitative variables. Student *t*-test or Mann-Whitney test were used for quantitative data. All results for continuous variables are described by using median and SD, or median and interquartile range (IQR). P-values less than or equal to 0.01 were considered statistically significant in the univariate analysis, whilst in multivariate analysis when P-values less than 0.05 were considered statistically significant. The association between dichotomous risk factors and sequelae was given by using chi-square test and calculating the Absolute Risk (AR), Relative Risk (RR) and their 95% Confidence Intervals (95%CI).

Two multiple logistic regression analyses were performed for sequelae in survivors related to risk factors on admission and risk factors during hospitalization. Multiple logistic regression analysis was performed using the backward conditional method to identify predictors of sequelae. Sequelae were used as dependent variables and candidated predictors selected when P-values were less than 0.05 in the univariate analysis. Moreover, we included in the logistic regression models variables which have been reported in the literature to be related with the prognosis of BM.

## Results

### Bacteriological findings

From 1974 to 2005 a total of 3,495 meningitis cases were recorded, out of which 2,477 were classified as bacterial in origin. A total of 1,146 cases (46.3%) were considered as probable BM while 1,331 cases (53.7%) were confirmed BM. The most common pathogens that were encountered were *Neisseria meningitidis *(838 cases, 63.0% of confirmed BM cases), Hib (252 cases, 18.9% of confirmed BM cases), *Streptococcous pneumoniae *(186 cases, 14.0% of confirmed BM cases) and 55 cases due to "other bacteria".

### Demographics

The demographics of the study population are presented in Table 1. The duration of symptoms before hospital admission varied significantly (p < 0.001) according to the pathogen of the BM. Patients with *N. meningitidis *and Hib presented earlier (median = 24.0 hrs, Interquartile range IQR = 20.0-48.0 hrs and median = 24.0 hrs, IQR = 24.0-72.0 hrs, respectively) compared to patients with meningitis due to *S. pneumoniae *or other bacteria (median = 48.0 hrs, IQR = 24.0-96.0 hrs and median = 48.0 hrs, IQR = 24.0-96.0 hrs, respectively). Prior to admission, 240 out of 2,477 patients (9.7%, 95%CI 8.6-11.0) had already received antibiotic therapy. In those patients the causative organism of BM was less commonly identified (RR 4.12, 95%CI 3.22-5.28, p < 0.001). Moreover, the duration of symptoms with respect to the three different periods (A, B, and C as defined earlier) showed to be statistically significant (p < 0.001). The duration of symptoms prior to hospital presentation was noticed to decrease from period A (Median = 24 hrs, IQR = 24-72 hrs) to period C (Median = 24 hrs, IQR = 18-48 hrs) (Table 2).

**Table 1:** Characteristics of the study population for all BM cases

Variable	N/Total* or Median (IQR)	Percentage (95% CI)	Missing (%)
Demographics			
Age (y)	2.0 (0.5-5.0)		71/2477 (2.9)
Male sex	1450/2471	58.7% (56.7-60.6)	6/2477 (0.2)
Duration of symptoms (hrs)	24.0 (24.0-48.0)		348/2477 (14.0)
Duration of symptoms < 24 h	473/2129	22.2% (20.5-24.1)	348/2477 (14.0)
Symptoms & signs at presentation			
Body temperature (°C)	39.0 (38.5-39.6)		1235/2477 (49.9)
Fever > 38	1158/1242	93.2% (91.7-94.5)	1235/2477 (49.9)
Headache^a^	871/1112	78.3% (75.8-80.7)	218/1330 (16.4)
Vomiting	1193/2074	57.5% (55.4-59.7)	403/2477 (16.3)
Meningeal signs^b^	1212/1477	82.1% (80.0-84.0)	214/1691 (12.7)
Haemorrhagic rash	967/2466	39.2% (37.3-41.2)	11/2477 (0.4)
Seizures	337/1777	19.0% (17.2-20.9)	700/2477 (28.3)
Bulging fontanelle^c^	416/918	45.3% (42.1-48.6)	300/1218 (24.6)
Grunting^c^	472/952	49.6% (46.4-52.8)	266/1218 (21.8)
Poor feeding	449/1611	27.9% (25.7-30.1)	866/2477 (35.0)
Shock	209/1689	12.4% (10.9-14.1)	788/2477 (31.8)
Coma	155/1671	9.3% (7.9-10.8)	806/2477 (32.5)
Indexes of inflammation in CSF at presentation			
White-cell count (cells/mm^3^)	1468.0 (268.5-5175.0)		84/2477 (3.4)
≤ 100 WBC/mm^3^	396/2,393	16.5% (15.1-18.1)	84/2477 (3.4)
≤ 1000 WBC/mm^3^	1063/2393	44.4% (42.4-46.4)	84/2477 (3.4)
% polymorphonuclears	88.0 (75.0-95.0)		507/2477 (20.5)
% lymphocytes	13.0 (6.0-27.0)		1282/2477 (51.8)
Glucose (mg/dl)	42.0 (18.25-58.0)		217/2477 (8.8)
Protein (mg/dl)	90.0 (42.0-190.0)		210/2477 (8.5)
Positive CSF culture	972/2082	46.7% (44.5-48.9)	395/2477 (15.9)
Positive CSF Gram stain	792/2022	39.2% (37.0-41.3)	455/2477 (18.4)
Indexes of inflammation in blood at presentation			
White-cell count (cells/mm^3^)	14000 (9500-19700)		186/2477 (7.5)
% polymorphonuclears	73.0 (58.0-84.0)		280/2477 (11.3)
% lymphocytes	19.0 (10.0-33.0)		456/2477 (18.4)
Positive Blood culture	284/1668	17.0% (15.3-18.9)	809/2477 (32.7)

* study population and denominators vary due to missing values.

a study population excludes children less than two years old (N = 1330)

b study population excludes children less than one year old (N = 1691)

c study population excludes children greater than or equal to two years old (N = 1218)

**Table 2:** Duration of symptoms according to periods

	Period									
	
	A			B			C			P-value*
		
	Mean	Median	IQR	Mean	Median	IQR	Mean	Median	IQR	
Duration of symptoms (in hours)	49.4	24	24-72	42.4	24	24-48	36.8	24	18-48	< 0.001

* Kruskal - Wallis test

Period A (1974-1984, pre Hib vaccination), Missing values: 149/1319 (11.3%)

Period B (1985-1994, Hib vaccine introduced), Missing values: 81/600 (13.5%)

Period C (1995-2005, post Hib vaccination), Missing values: 118/558 (21.1%)

### Clinical data

As shown in Table 1 the vast majority of cases at initial presentation were febrile (93.2%), with an estimated median body temperature of 39.0°C (IQR = 38.5-39.6). Meningeal signs in children greater than or equal to one year of age were present in 82.1% of patients on admission. Out of 838 patients with confirmed meningococcal meningitis 511 patients (61.0%) presented with hemorrhagic rash. Hemorrhagic rash was also one of the presenting symptoms in 17 out of 186 (9.2%) patients with meningitis due to *S. pneumoniae *and 17 out of 252 (6.7%) patients with Hib meningitis. The frequency of different clinical signs and symptoms of all registered bacterial meningitis cases on admission are presented in Table 1 in conjunction with laboratory findings and other parameters.

Clinical manifestations of bacterial meningitis were found to be dependent on the age of the patient, as shown in Table 3. The most common findings in infants included grunting (57.6%, RR 1.64, 95%CI 1.38-1.97), poor feeding (54.7%, RR 4.93, 95%CI 4.06-5.97) and bulging fontanelle (58.2%, RR 3.70, 95%CI 2.73-5.01). Seizures were more common in children under one year of age compared to older children (RR 1.70, 95%CI 1.40-2.06), while infants presented less frequently with coma (RR 0.49, 95%CI 0.33-0.73). Fever however, was found to be independent of age with frequencies of fever > 90% throughout all ages as seen in Table 3.

**Table 3:** Variations of clinical manifestations according to age

	N/Total (%)								
	
Clinical Features	Age < 1 y (n = 786)		Age 1-4 y (n = 932)		Age 5-9 y (n = 480)		Age > = 10 y (n = 208)		P- value
**All cases**									
Fever	383/408	(93.9)	421/459	(91.7)	225/236	(95.3)	101/109	(92.7)	0.303
Shock	66/541	(12.2)	84/646	(13.0)	35/317	(11.0)	18/140	(12.9)	0.849
Coma	29/525	(5.5)	58/639	(9.1)	33/317	(10.4)	32/145	(22.1)	< 0.001
Bulging Fontanelle^a^	371/637	(58.2)	37/235	(15.7)	-	-	-	-	< 0.001
Grunting^a^	372/646	(57.6)	91/260	(35.0)	-	-	-	-	< 0.001
Poor feeding	330/603	(54.7)	103/585	(17.6)	0/257	(0.0)	4/121	(3.3)	< 0.001
Seizures	155/600	(25.8)	134/678	(19.8)	24/316	(7.6)	14/137	(10.2)	< 0.001
Headache^b^	-	-	304/447	(68.0)	369/424	(87.0)	167/189	(88.4)	< 0.001
Meningeal signs^c^	-	-	655/811	(80.8)	360/426	(84.5)	151/182	(83.0)	0.252
Haemorrhagic rash	225/786	(28.6)	452/930	(48.6)	190/479	(39.7)	76/207	(36.7)	< 0.001
Vomiting	269/622	(43.2)	477/792	(60.2)	289/424	(68.2)	123/179	(68.7)	< 0.001
**Confirmed cases**									
Fever	218/231	(94.4)	270/286	(94.4)	102/108	(94.4)	44/46	(95.7)	0.988
Shock	40/312	(12.8)	48/397	(12.1)	20/144	(13.9)	6/56	(10.7)	0.918
Coma	18/301	(6.0)	37/394	(9.4)	24/148	(16.2)	14/57	(24.6)	< 0.001
Bulging Fontanelle^a^	242/359	(67.4)	23/159	(14.5)	-	-	-	-	< 0.001
Grunting^a^	243/369	(65.9)	66/185	(35.7)	-	-	-	-	< 0.001
Poor feeding	205/339	(60.5)	77/361	(21.3)	0/117	(0.0)	3/46	(6.5)	< 0.001
Seizures	93/343	(27.4)	86/418	(20.6)	11/142	(7.7)	7/54	(13.0)	< 0.001
Headache^b^	-	-	174/250	(69.6)	157/183	(85.8)	66/75	(88.0)	< 0.001
Meningeal signs^c^	-	-	414/501	(82.6)	148/183	(80.9)	55/70	(78.6)	0.660
Haemorrhagic rash	119/440	(27.0)	274/570	(48.1)	102/204	(50.0)	39/80	(48.8)	< 0.001
Vomiting	167/357	(46.8)	310/490	(63.3)	128/184	(69.6)	53/72	(73.6)	< 0.001

a Study population excludes children ≥ 2 years old

b Study population excludes children < 2 years old

c Study population excludes children < 1 years old

There were 71 missing values in Age

Missing values ranged from 0% to 37.2%

Older children (≥ 1 years old to four years old) presented with fever (91.7%) headache (68.0%), meningeal signs (80.8%), hemorrhagic rash (48.6%) and vomiting (60.2%). Similar frequencies were noticed for age groups five to nine years, and greater than ten years old inclusive. Similar frequencies were noticed when analyzing "confirmed BM cases" to those of "All BM cases" for all age groups. Fever was found to be independent of age even in the confirmed BM population, as seen in Table 3.

Changes in clinical signs and symptoms have also been observed during the three decades of the study. Examination over the three different study periods (A, B and C respectively) revealed a statistically significant reduction in the percentage of patients that presented with meningeal signs (80.7%, 72.6% and 51.3%, p < 0.001), seizures (24.8%, 16.0% and 9.4%, p < 0.001), bulging fontanelle (34.0%, 25.5% and 8.5%, p < 0.001), grunting (38.0%, 34.8% and 14.4%, p < 0.001), shock (18.6%, 5.9% and 5.9%, p < 0.001) and coma (11.7%, 7.3% and 6.2%, p = 0.002). On the contrary the rate of hemorrhagic rash (39.1%, 34.8% and 44.1%, p = 0.006) increased significantly in period C.

### Laboratory findings

The laboratory profile of children with BM is presented in Table 1. The results of laboratory findings and the analyses that followed were performed on data extracted from the Meningitis Registry, where often physicians may not have fully completed the registry forms, therefore creating missing values in our analyses. The median value of CSF WBC on admission was 1,468.0/mm^3 ^(IQR = 268.5 - 5,175.0), while 1,063 out of 2,393 BM cases (44.4%, 95%CI 42.4 - 46.4) having less than 1,000 CSF WBC/mm^3^. A total of 198 confirmed BM cases had less than or equal to 100 CSF WBC/mm^3 ^of which *N. meningitidis *were isolated in 85.9%, *S. pneumoniae *in 8.1%, Hib in 1.5% and other bacteria in 4.5% of those patients. On admission median CSF glucose was estimated at 42.0 mg/dl (IQR = 18.5-58.0) with a median CSF protein value of 90.0 mg/dl (IQR = 42.0-190.0) (Table 1).

### Sequelae and complications

The case fatality rate of BM for the entire study population from all causes was estimated to be 3.8% (95/2,477, 95%CI 3.1-4.7) [3]. Case fatality rates with respect to age groups and periods have been previously discussed in detail [3]. The estimated case fatality rate from probable N.*meningitidis *cases was 11.1% (95%CI 8.3-14.6) while only 1.1% (95%CI 1.0-2.9) was estimated for confirmed cases of N.*meningitidis*.

The rate of sequelae among survivors was estimated 3.3% (83 episodes occurred in 73/2,207 BM cases, 95%CI 2.6-4.2). From our study, only one child out of 2,207 (0.04%) patients survived BM with major disabilities (quadriplegia and mental retardation).

The rate of acute complications was estimated 6.8% (152/2,251 BM cases, 95% CI 5.8-7.9) with a total of 153 episodes of complications in 152 meningitis cases. As shown in additional file 1 arthritis and subdural effusion were the most frequent complications (3.6% and 3.2% respectively). The frequency of acute complications and sequelae according to the causative agent of BM is presented in detail in additional file 1. Severe hearing loss was diagnosed in 4 out of 160 (2.5%, 95% CI 0.7-6.3) BM cases with pneumococcal meningitis, 7 out of 247 (2.8%, 95% CI 1.1-5.8) BM cases with Hib meningitis, 4 out of 1,104 (0.4%, 95% CI 0.1-1.0) BM cases with meningococcal meningitis and 1 out of 45 (2.2%, 95% CI 0.1-11.8) BM cases with meningitis due to other bacteria. No difference was identified regarding sequelae occurrence in the three time periods of the study (p = 0.410).

### Prognostic factors

Analyses of the association of various clinical and laboratory factors with sequelae are shown in Tables 4, 5 and 6. Analysis of prognostic factors with respect to "all BM cases" and "confirmed BM cases" showed similar statistical significance for all prognostic factors as shown in Table 4 with the exception of CSF WBC ≤ 1000/μL and positive CSF culture which showed significance only for BM cases and not for confirmed BM cases. However, when positive CSF culture and CSF ≤ 1000/ul were included in a multivariate analysis, no statistical significance was noted. Moreover, as seen in Table 6 poor feeding with respect to sequelae showed to be statistically significant only for BM cases and not for confirmed BM cases.

**Table 4:** Univariate analysis of CSF and peripheral blood as prognostic factors for sequelae in childhood bacterial meningitis

		All BM cases (n = 2382)					Confirmed cases (n = 1297)				
		
Prognostic factors		Sequelae/Prognostic factor* (%)		RR	(95% CI)	P-value	Sequelae/Prognostic factor* (%)		RR	(95% CI)	P-value
CSF fluid analysis at presentation											
Positive CSF culture	Yes	41/904	(4.5)	2.18	(1.30-3.66)	**0.002**	41/901	(4.5)	1.41	(0.64-3.10)	0.387
	No	21/1009	(2.1)				7/217	(3.2)			
Positive Gram stain	Yes	41/732	(5.6)	2.86	(1.72-4.76)	**< 0.001**	40/725	(5.5)	2.05	(1.01-4.18)	0.041
	No	22/1123	(2.0)				9/335	(2.7)			
CSF WBC ≤ 100/μL	Yes	8/341	(2.3)	0.67	(0.32-1.37)	0.172	5/175	(2.9)	0.59	(0.24-1.45)	0.237
	No	65/1843	(3.5)				50/1024	(4.9)			
CSF WBC ≤ 1000/μL	Yes	24/945	(2.5)	0.64	(0.40-1.04)	0.043	16/383	(4.2)	0.87	(0.49-1.54)	0.642
	No	49/1239	(4.0)				39/816	(4.8)			
CSF Glu < 40 mg/dL	Yes	57/949	(6.0)	5.53	(2.98-0.24)	**< 0.001**	46/665	(6.9)	4.81	(2.19-10.57)	**< 0.001**
	No	12/1104	(1.1)				7/487	(1.4)			
CSF Protein > 100 mg/dL	Yes	52/987	(5.3)	3.34	(1.95-5.74)	**< 0.001**	43/646	(6.7)	3.31	(1.68-6.53)	**< 0.001**
	No	17/1078	(1.6)				10/498	(2.0)			
≥ 65% neutrophils in CSF	Yes	57/1534	(3.7)	1.75	(0.76-4.01)	0.119	44/911	(4.8)	1.43	(0.45-4.52)	0.383
	No	6/282	(2.1)				3/89	(3.4)			
Peripheral blood analysis at presentation											
Positive blood culture	Yes	18/256	(7.0)	3.11	(1.76-5.52)	**< 0.001**	18/251	(7.2)	2.72	(1.41-5.25)	**0.002**
	No	29/1284	(2.3)				16/607	(2.6)			
Peripheral WBC < 5000/μL	Yes	3/104	(2.9)	0.85	(0.27-2.66)	0.533	3/83	(3.6)	0.77	(0.24-2.41)	0.455
	No	68/2009	(3.4)				51/1084	(4.7)			
Peripheral WBC ≥ 15000/μL	Yes	26/921	(2.8)	0.75	(0.47-1.20)	0.139	18/505	(3.6)	0.66	(0.38-1.14)	0.131
	No	45/1192	(3.8)				36/662	(5.4)			
Absolute blood neutrophil count < 1000/μL	Yes	1/13	(7.7)	2.38	(0.36-5.92)	0.350	1/11	(9.1)	2.04	(0.31-13.49)	0.399
	No	64/1984	(3.2)				49/1100	(4.5)			

* Study population varies due to missing values

Missing values ranged from 8.3% to 35.3%

**Table 5:** Univariate analysis of prognostic factors for sequelae in childhood BM

		All BM cases (n = 2382)					Confirmed cases (n = 1297)				
	
Prognostic Factors		Sequelae/Prognostic factor*	(%)	RR	(95% CI)	P-value	Sequelae/Prognostic factor*	(%)	RR	(95% CI)	P-value
Etiology											
*N. meningitidis*	Yes	15/1091	(1.4)	0.26	(0.15-0.46)	**< 0.001**	15/763	(2.0)	0.22	(0.12-0.39)	**< 0.001**
	No	58/1116	(5.2)				40/445	(9.0)			
*S. pneumoniae*	Yes	20/161	(12.4)	4.80	(2.94-7.82)	**< 0.001**	20/161	(12.4)	3.72	(2.20-6.27)	**< 0.001**
	No	53/2046	(2.6)				35/1047	(3.3)			
*H. influenzae*	Yes	11/240	(4.6)	1.45	(0.78-2.72)	0.163	11/240	(4.6)	1.01	(0.53-1.92)	0.980
	No	62/1967	(3.2)				44/968	(4.5)			
Other bacteria	Yes	9/44	(20.5)	6.91	(3.68-12.99)	**< 0.001**	9/44	(20.5)	5.18	(2.71-9.89)	**< 0.001**
	No	64/2163	(3.0)				46/1164	(4.0)			
Unknown	Yes	18/671	(2.7)	0.75	(0.44-1.27)	0.170	-	-	-	-	**-**
	No	55/1536	(3.6)				-	-			
Various Characteristics											
Male sex	Yes	45/1296	(3.5)	1.12	(0.71-1.79)	0.356	33/690	(4.8)	1.12	(0.66-1.90)	0.669
	No	28/907	(3.1)				22/516	(4.3)			
Duration of symptoms < 24 h	Yes	5/423	(1.2)	0.32	(0.13-0.80)	**0.004**	3/240	(1.3)	0.24	(0.07-0.75)	**0.007**
	No	56/1524	(3.7)				44/828	(5.3)			
Age < 2 y	Yes	50/1038	(4.8)	2.43	(1.49-3.99)	**< 0.001**	39/624	(6.3)	2.15	(1.22-3.81)	**0.007**
	No	22/1112	(2.0)				16/551	(2.9)			
Period C	Yes	11/384	(2.9)	0.84	(0.45-1.58)	0.364	11/247	(4.5)	0.97	(0.51-1.86)	0.933
	No	62/1823	(3.4)				44/961	(4.6)			

* Prognostic factor population varies due to missing values

Missing values ranged from 7.3% to 18.3%

**Table 6:** Univariate analysis of prognostic factors for sequelae of all cases and confirmed BM cases

		All BM cases (n = 2382)					Confirmed cases (n = 1297)				
	
Prognostic Factors		Sequelae/prognostic factor*	(%)	RR	(95% CI)	P-value	Sequelae/prognostic factor*	(%)	RR	(95% CI)	P-value
Clinical features at presentation											
Fever ≥ 38°C	Yes	33/1067	(3.1)	0.53	(0.19-1.46)	0.182	29/607	(4.8)	0.54	(0.17-1.69)	0.237
	No	4/69	(5.8)				3/34	(8.8)			
Headache ^a^	Yes	16/796	(2.0)	0.90	(0.33-2.42)	0.501	12/386	(3.1)	1.19	(0.34-4.15)	0.536
	No	5/223	(2.2)				3/115	(2.6)			
Vomiting	Yes	25/1102	(2.3)	0.50	(0.30-0.82)	**0.004**	17/631	(2.7)	0.38	(0.21-0.68)	**< 0.001**
	No	37/808	(4.6)				30/422	(7.1)			
Meningeal	Yes	22/1120	(2.0)	0.79	(0.32-1.92)	0.372	14/601	(2.3)	0.61	(0.23-1.68)	0.246
signs ^b^	No	6/240	(2.5)				5/132	(3.8)			
Haemorrhagic	Yes	7/859	(0.8)	0.17	(0.08-0.36)	**< 0.001**	6/505	(1.2)	0.17	(0.07-0.39)	**< 0.001**
rash	No	66/1348	(4.9)				49/703	(7.0)			
Seizures	Yes	33/299	(11.0)	4.61	(2.88-7.38)	**< 0.001**	25/182	(13.7)	4.03	(2.37-6.85)	**< 0.001**
	No	32/1338	(2.4)				25/734	(3.4)			
Bulging	Yes	33/390	(8.5)	2.80	(1.52-5.15)	**< 0.001**	26/256	(10.2)	2.32	(1.17-4.59)	0.012
fontanelle ^c^	No	14/463	(3.0)				11/251	(4.4)			
Grunting ^c^	Yes	30/422	(7.1)	2.14	(1.17-3.92)	0.011	25/282	(8.9)	2.00	(1.00-3.98)	0.043
	No	15/452	(3.3)				11/248	(4.4)			
Poor feeding	Yes	25/415	(6.0)	1.92	(1.16-3.17)	**0.010**	20/274	(7.3)	1.57	(0.89-2.76)	0.117
	No	34/1081	(3.1)				26/558	(4.7)			
Shock	Yes	8/166	(4.8)	1.37	(0.66-2.84)	0.256	6/104	(5.8)	1.17	(0.51-2.71)	0.710
	No	49/1392	(3.5)				38/772	(4.9)			
Coma	Yes	7/116	(6.0)	1.76	(0.81-3.79)	0.122	6/79	(7.6)	1.62	(0.71-3.73)	0.187
	No	49/1427	(3.4)				37/791	(4.7)			
Clinical features at day 3											
Fever	Yes	29/465	(6.2)	2.65	(1.63-4.32)	**< 0.001**	24/305	(7.9)	2.65	(1.50-4.68)	**< 0.001**
	No	33/1404	(2.4)				21/707	(3.0)			
Headache ^a^	Yes	4/119	(3.4)	3.08	(0.94-10.07)	0.073	3/64	(4.7)	5.5	(1.14-26.65)	0.049
	No	8/733	(1.1)				3/352	(0.9)			
Vomiting	Yes	2/22	(9.1)	3.13	(0.81-12.09)	0.139	2/15	(13.3)	3.63	(0.95-13.79)	0.110
	No	45/1548	(2.9)				31/843	(3.7)			
Meningeal	Yes	9/252	(3.6)	3.05	(1.25-7.41)	0.015	5/141	(3.5)	3.16	(0.93-10.77)	0.066
signs ^b^	No	10/853	(1.2)				5/446	(1.1)			
Haemorrhagic	Yes	1/264	(0.4)	0.11	(0.02-0.79)	**0.002**	1/152	(0.7)	0.15	(0.02-1.06)	0.025
rash	No	42/1209	(3.5)				30/666	(4.5)			
Seizures	Yes	23/71	(32.4)	12.75	(7.89-20.59)	**< 0.001**	17/49	(34.7)	10.51	(6.03-18.33)	**< 0.001**
	No	32/1259	(2.5)				23/697	(3.3)			
Bulging	Yes	19/109	(17.4)	4.77	(2.67-8.51)	**< 0.001**	13/78	(16.7)	2.86	(1.49-5.49)	**0.001**
fontanelle ^c^	No	22/602	(3.7)				20/343	(5.8)			
Grunting ^c^	Yes	7/25	(28.0)	6.21	(3.03-12.72)	**< 0.001**	5/21	(23.8)	3.75	(1.60-8.77)	0.012
	No	31/688	(4.5)				26/409	(6.4)			
Poor feeding	Yes	8/28	(28.6)	9.37	(4.80-18.27)	**< 0.001**	7/24	(29.2)	7.26	(3.50-15.04)	**< 0.001**
	No	36/1180	(3.1)				26/647	(4.0)			
Shock	Yes	0/9	(0.0)	2.91	(0.44-19.10)	0.300	0/6	(0.0)	3.22	(0.51-20.42)	0.278
	No	43/1250	(3.4)				31/699	(4.4)			
Coma	Yes	5/13	(38.5)	12.17	(5.73-25.86)	**< 0.001**	5/11	(45.5)	11.6	(5.50-24.45)	**< 0.001**
	No	39/1234	(3.2)				27/689	(3.9)			

^a ^Study population excludes children < 2 years old, ^b ^Study population excludes children < 1 year old, ^c ^study population excludes children ≥ 2 years old,

* Prognostic factor population varies due to missing values

Missing values ranged from 7.3% to 38.8%

The results of multiple logistic regression analysis are presented in Table 7. Findings on admission including seizures, absence of hemorrhagic rash, low CSF glucose, high CSF protein, positive blood culture and either pneumococcal or meningococcal meningitis were found to be independent predictors of sequelae in survivors of BM.

**Table 7:** Multivariate analysis of prognostic factors for sequelae in childhood bacterial meningitis

Prognostic factors	OR	95% CI	P value
Risk factors at presentation			
Seizures	5.36	2.63-10.90	< 0.001
Hemorrhagic rash	0.21	0.06-0.69	0.011
CSF Glucose < 40 mg/dl	5.18	1.69-15.87	0.004
CSF Protein > 100 mg/dl	2.97	1.06-8.29	0.038
Positive blood culture	2.66	1.24-5.72	0.012

Note: this analysis is based on 44.7% of the study population due to missing values

**Figure 1:**
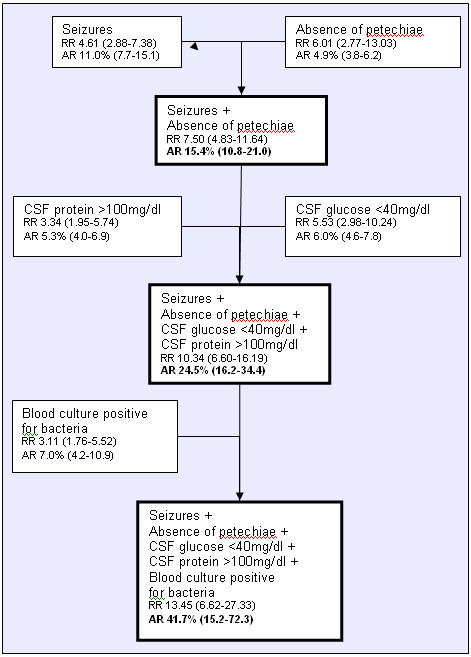
**Absolute and relative risk for sequelae according to findings on admission**.

Prognostic algorithms were created using risk factors identified in the multivariate logistic regression analysis. Absolute and relative risk (RR) for sequelae were also calculated. Children presenting with seizures, absence of petechiae, low CSF glucose and a high CSF protein had an absolute risk for sequelae of 24.5% (95%CI 16.2-34.4). While children presenting with the above mentioned prognostic factors in addition to positive blood cultures had an absolute risk for sequelae of 41.7% (95%CI 15.2-72.3) (Figure 1).

Furthermore, each acute complication or sequelae was examined individually for the identification of independent risk factors. Various parameters, such as causative organism, age of patient, sex, duration of symptoms before admission and chronological period, were analyzed with respect to occurrence of arthritis, subdural effusion, ventriculitis, hydrocephalus, severe hearing loss, and seizure disorder. Analysis of seizures on admission were found to be strongly associated with the development of seizure disorder as a sequelae (RR = 8.02, 95%CI 3.40-18.96, p < 0.001), while the RR increased to 24.77 (95%CI 10.29-59.65, p < 0.001) when seizures occurred on the 3rd day.

Subsequently, multiple logistic regression analysis of prognostic factors was performed for each acute complication and sequelae. It was found that meningitis due to *S. pneumoniae *(OR 4.7, 95%CI 2.5-8.8), age < 1 year (OR 18.0, 95%CI 7.7-42.3) and duration of symptoms > 24 hours (OR 2.1, 95% CI 1.2-3.8) are independent risk factors for subdural effusion. Meningitis due to bacteria other than *N. meningitidis, S. pneumoniae *and Hib (OR 15.4, 95%CI 5.7-41.4) and age < 1 year (OR 3.2, 95%CI 1.3-8.2) were found to be related with ventriculitis. Arthritis was more common in meningococcal meningitis (OR 2.8, 95%CI 1.7-4.8), while pneumococcal meningitis (OR 6.0, 95%CI 1.9-19.0) and age < 1 year (OR 9.8, 95%CI 2.1-44.5) were independent risk factors for hydrocephalus. The same risk factors, pneumococcal meningitis (OR 12.8, 95% CI 5.6-29.0) and age < 1 year (OR 13.7, 95%CI 4.0-46.2) were independently related with seizure disorder.

Severe hearing loss was more common in patients with Hib (OR 3.6, 95%CI 1.5-8.5), and pneumococcal meningitis (OR 2.7, 95%CI 0.92-7.8; p = 0.08) although results were not statistically significant). On the other hand severe hearing loss was less common yet statistically significant in patients with *N. meningitidis *(OR 0.2, 95%CI 0.07-0.7). Although analysis of time period showed to be significant for some sequelae, it was not found to be an independent risk factor in multivariate analysis.

The current study highlights various prognostic factors of sequelae such as positive blood culture, bulging fontanelle, and poor feeding that have not yet been dealt before in previous studies.

## Discussion

The present study examined clinical, laboratory and general characteristics of children with acute BM in order to identify independent predictors of sequelae. Currently presented information has been extracted from an established meningitis registry derived from an extensive series of patients collected prospectively over a period of 32 years in a tertiary children's hospital [3].

Findings regarding epidemiological trends with respect to etiology of BM, from 1974 to 2005 in Greece, have been discussed extensively elsewhere [3]. From our study, clinico-laboratory findings of these patients largely support what is currently known about childhood BM [8]. Presenting signs and symptoms depended significantly on the age of the patient. Infants less than one years of age presented more commonly with seizures, bulging fontanelle, grunting and poor feeding as expected for such an age group. However this age group presented less commonly with coma. Toddlers and children greater than two years of age manifested more often with meningeal signs, headache, vomiting and haemorrhagic rash. Fever however was common for all age groups, therefore it can be concluded that fever is independent of age. It would also be feasible to suggest that our population with fever may be somewhat lower than it should be due to the admission of antipyretics by parents prior to hospital admission.

The introduction of the Hib vaccine at the end of period B is apparent as the rate of children presenting with seizures, bulging fontanelle, grunting, meningeal signs, shock and coma reduced significantly in period C compared to periods A and B. Improved socioeconomic conditions resulted in increased public awareness about the disease, improved availability of medical services, early recognition of signs and symptoms by caregivers and health professionals and prompt referral to tertiary medical centres. All of these changes are reflected in a reduction by more than 12 hours of the interval between the onset of symptoms to hospital admission, from period A to period C in the current study. When considering our study group, 71.3% of Hib meningitis cases were < 2 years of age, while patients < 5 years of age showed a reduction of 92.5% for the incidence rate of Hib meningitis with the introduction of Hib conjugate vaccine during period C (post Hib era) [3]. Hence, it can be concluded that the reduction in the rates of seizures, bulging fontanelle and grunting - all related to infantile meningitis - is largely attributed to the introduction and usage of Hib conjugate vaccine. Moreover, an outbreak of serogroup C meningococcal disease that occurred in Greece in the years 1996-1997 increased the mean age of children affected from BM during period C, and was also responsible for the increased rate of haemorrhagic rash in the same period [9].

The case fatality rate estimated in the present study of 3.8% is comparable to rates from other developed countries (4.8%) as reported in the meta-analysis by Baraff et al [10]. On the other hand, the currently estimated rate of sequelae overall (3.3%), is lower when compared to other reports in the literature, which ranges from 8% to 37% - with an average around 15% to 17.5% [1,11-16]. Such a variation may be attributed to the differences in the criteria adopted in each study in the selection of its study population defined by e.g. age, pathogen type, disease severity and even definition of sequelae. Nevertheless, we believe that the sequelae rates estimated in our study are possibly underestimated, due to incomplete update of the registry forms by the attending physicians, which is a known weakness of disease registries. The present study supports the current literature with respect to sequelae being less common in meningococcal meningitis. *N.meningitis *in our study showed to be more common but with lower rates of sequelae. This leads to an underestimation of sequelae in our series when compared to other studies where Hib was predominant cause of meningitis. This could partially explain the lower estimates of sequelae rates in our study.

Another factor that might have influenced the low rate of sequelae in the present study is the extensive use of corticosteroids in our hospital for BM from the mid 70s, almost 20 years before the first prospective double-blind studies that supported the use of dexamethasone in childhood BM [17].

According to previous reports, hearing impairment of any type is expected to develop in 5.6% to 30.6% of patients with bacterial meningitis, while severe, profound or bilateral hearing loss is expected to occur in 1.0 to 5.1% of patients [10,14,18-21]. These findings are in agreement with the current study as 23 out of 2,235 children (1.0%), developed severe hearing loss.

Analyses of a large number of variables were found to correlate with sequelae. Some of these variables that have not been previously reported include the absence of headache (related with septicemia and meningitis rather than meningitis alone), bulging fontanelle, grunting and poor feeding on admission (physical signs encountered in infants), vomiting on admission and a positive blood culture or CSF gram stain. Moreover, presence of meningeal signs, bulging fontanelle, grunting or poor feeding on the 3^rd ^day of hospitalization (indicating the occurrence of complications or sequelae such as ventriculitis or hydrocephalus) or absence of petechiae on the 3^rd ^day could be used as prognostic factors.

Furthermore, multivariate statistical analysis was applied in order to control the confounding influence of other variables and to assess the independent effect of risk factors on the outcome. Presence of seizure on admission and on the 3^rd ^post-admission day increased the risk of sequelae significantly (OR = 5.36 and OR = 17.80 respectively). Many authors report similar findings, while some correlate atypical convulsions with later sequelae [5,11,12,16,22-24]. According to our findings the occurrence of seizures on admission were strongly associated with the development of seizure disorder as a sequelae (RR = 4.61), while the RR increased to 12.75 when seizures occurred on the 3^rd ^day. Current data demonstrate that late seizures are stronger predictors of seizure disorder.

Low CSF glucose was found to be an important prognostic factor for the development of sequelae (OR = 5.18), in agreement with other studies that correlated low CSF glucose with increased risk of hearing loss, with increased risk of death or epilepsy, or with lower ratio of CSF and blood glucose with increased risk of sequelae [6,23,25]. High CSF protein considered to be an indicator of CSF inflammation, was found to independently predict the occurrence of sequelae (OR = 3.97), as previously reported [16]. Positive blood culture is reported for the first time as a prognostic factor for sequelae in children suffering from BM (OR = 2.66). Blood cultures are not considered to be extremely invasive to patients and results are available during the first few days of hospital stay.

On multivariate analysis, either isolation of *N. Meningitidis *or presence of hemorrhagic rash were predictors of a favourable outcome regarding sequelae. Similar findings have been reported in a study on risk factors for meningococcal disease, another on the prognosis of hearing loss, while Biesheuvel et al. incorporated the absence of petechiae in a prediction rule for neurological sequelae after childhood bacterial meningitis [5,6]. Similarly the role of pneumococcal meningitis as a prognostic factor for sequelae in BM has also been well documented [5,6,12]. No independent relationship was found between young age, duration of symptoms, presence of shock or CSF WBC and sequelae.

In the present study certain combinations of prognostic factors such as seizures, absence of petechiae, low CSF glucose, high CSF protein and a positive blood culture have a significantly increased risk for sequelae. Similarly, absence of petechiae/ecchymoses and use of anti-epileptics for > 2 days have been used in a prognostic scoring system for sequelae in childhood BM [5].

Although current study focuses on short term effects of BM in children, several studies have pointed out the presence of long term neurobehavioural effects. Academic, intellectual and executive ability may be negatively influenced in these children, as they are found to perform worse compared to their peers [11,26].

Our study concentrates on the prognostic factors associated with meningitis disease in Greece only. One of the main advantages of this study is that, by engaging a total of 2,477 children, it turns out to be one of the biggest cohorts of children affected with bacterial meningitis. Such a high number of patients allows for safe conclusions to be drawn, supported by relatively narrow confidence intervals in the statistical analysis. Moreover, due to the extended period that the meningitis was in operation, we were able to delineate long-term changes in the clinical and laboratory characteristics of childhood BM. On the other hand, long duration of the study was related with one limitation being the lack of homogeneity in the data of the registry forms. The use of antibiotic therapy prior to bacterial confirmation of meningitis may also affect the results of this study giving an under-representation of confirmed cases. Moreover, a possible limitation of the study is the percentage of missing values ranging from 0.4% to 35% in different variables without having any indication that they were created systematically. However, we have to acknowledge that missing values is a weakness of the analysis.

## Conclusion

In conclusion, current data indicate that sequelae can be predicted by a combination of seizures, absence of hemorrhagic rash, low CSF glucose, high CSF protein and a positive blood culture. Etiology is critical for the prognosis of sequelae in childhood BM. Late findings, including a combination of bulging fontanelle and seizures on the 3^rd ^day also correlate with increased sequelae. These risk factors may be of value in selecting patients for more intensive therapy and in identifying possible candidates for new treatment strategies.

## Abbreviations

AR: absolute risk; BM: bacterial meningitis; CI: confidence intervals; CFR: case fatality rate; CRP: C-reactive protein; CSF: cerebrospinal fluid; CT: computed tomography; GCS: Glascow Coma Scale; Hib: *Haemophilus influenza *type b; IQR: interquartile range; IR: incidence rate; MR: meningitis registry; OR: odds ratio; RF: registry form; RR: relative risk; SD: standard deviation

## Competing interests

The authors declare that they have no competing interests.

## Authors' contributions

MNT and CSH designed the study. MNT, VAV and ATK collected the data. CSH and VAV performed the statistical analysis. MNT, VAV and CSH interpreted the results. MNT, VAV, MK wrote the manuscript. KT provided valuable insight for revising the manuscript. All authors read and approved the final manuscript. CSH is the guarantor.

## Acknowledgements

We would like to thank all pediatricians who participated in the collection of data by filling the Registry Form. Moreover, we would like to thank Dr J Economides who performed hearing screening in children with bacterial meningitis.

## Pre-publication history

The pre-publication history for this paper can be accessed here:

http://www.biomedcentral.com/1471-2334/11/214/prepub

## Supplementary Material

Additional file 1 **Frequency of acute complications and sequelae in survivors of BM according to etiology**. Click here for file
