# Supplementary material for: Prognostic factors related to sequelae in childhood bacterial meningitis: Data from a Greek meningitis registry
Source: BMC Infect Dis. 2011 Aug 10;11:214. doi: 10.1186/1471-2334-11-214 (PMC3166933; doi:10.1186/1471-2334-11-214)
Supplement: Additional file 1 — Frequency of acute complications and sequelae in survivors of BM according to etiology. [file 1471-2334-11-214-S1.DOC]

Additional file 1. Frequency of acute complications and sequelae in survivors of BM according to etiology

| **Etiology** | **Acute complications** | | | | **Sequelae** | | | | | | | |
| --- | --- | --- | --- | --- | --- | --- | --- | --- | --- | --- | --- | --- |
|  | **Arthritis** | | **Subdural Effusion** | | **Ventriculitis** | | **Hydrocephalus** | | **Severe Hearing Loss** | | **Seizure Disorder** | |
|  | **N/Total** | **%**  **(95% CI)** | **N/Total** | **%**  **(95% CI)** | **N/Total** | **%**  **(95%CI)** | **N/Total** | **%**  **(95% CI)** | **N/Total** | **%**  **(95% CI)** | **N/Total** | **%**  **(95% CI)** |
| *N. meningitidis* | 60/1,121 | 5.4  (4.1-6.9) | 19/1,118 | 1.7  (1.1-2.7) | 4/1128 | 0.4  (0.1-1.0) | 2/1116 | 0.2  (0.0-0.7) | 4/1104 | 0.4  (0.1-1.0) | 6/1,117 | 0.5  (0.2-1.2) |
| *S. pneumoniae* | 2/161 | 1.2  (0.2-4.4) | 20/162 | 12.3  (7.7-18.4) | 5/166 | 3.0  (1.0-6.9) | 4/161 | 2.5  (0.7-6.2) | 4/160 | 2.5  (0.7-6.3) | 12/164 | 7.3  (3.8-12.4) |
| *H. influenzae* | 7/240 | 2.9  (1.2-5.9) | 16/240 | 6.7  (3.9-10.6) | 2/242 | 0.8  (0.1-3.0) | 0/240 | 0.0  (0.0-1.5) | 7/247 | 2.8  (1.1-5.8) | 2/240 | 0.8  (0.1-3.0) |
| Other | 4/45 | 8.9  (2.5-21.2) | 9/45 | 20.0  (9.6-34.6) | 7/47 | 14.9  (6.2-28.3) | 2/44 | 4.5  (0.6-15.5) | 1/45 | 2.2  (0.1-11.8) | 2/45 | 4.4  (0.5-15.1) |
| Unknown | 8/682 | 1.2  (0.5-2.4) | 8/682 | 1.2  (0.5-2.4) | 6/686 | 0.9  (0.4-2.0) | 4/682 | 0.6  (0.2-1.6) | 7/679 | 1.0  (0.5-2.2) | 2/681 | 0.3  (0.1-1.2) |
| TOTAL | 81/2,249 | 3.6  (2.9-4.5) | 72/2,247 | 3.2  (2.5-4.0) | 22/2,243 | 1.0  (0.6-1.5) | 12/2,243 | 0.5  (0.3-1.0) | 23/2,235 | 1.0  (0.7-1.6) | 24/2,247 | 1.1  (0.7-1.6) |
